# FCR方案一线治疗慢性淋巴细胞白血病/小淋巴细胞淋巴瘤的疗效及长期随访情况

**DOI:** 10.3760/cma.j.cn121090-20250107-00013

**Published:** 2025-11

**Authors:** 萧 路, 奕 夏, 祎 缪, 彤璐 邱, 罗梦佳 戴, 子元 周, 晖 金, 海荣 仇, 纯 乔, 雨洁 吴, 磊 范, 卫 徐, 建勇 李, 华渊 朱

**Affiliations:** 1 南京医科大学第一附属医院，江苏省人民医院血液科淋巴瘤中心，南京 210029 Lymphoma Center, Department of Hematology, Jiangsu Province Hospital, The First Affiliated Hospital of Nanjing Medical University, Nanjing 210029, China; 2 南京医科大学血液研究重点实验室，南京 210029 Key Laboratory of Hematology of Nanjing Medical University, Nanjing 210029, China

**Keywords:** 慢性淋巴细胞白血病, 氟达拉滨, 环磷酰胺, 利妥昔单抗, 布鲁顿酪氨酸激酶抑制剂, Chronic lymphocytic leukemia, Fludarabine, Cyclophosphamide, Rituximab, Bruton tyrosine kinase inhibitors

## Abstract

**目的:**

探讨FCR（氟达拉滨+环磷酰胺+利妥昔单抗）方案一线治疗慢性淋巴细胞白血病/小淋巴细胞淋巴瘤（CLL/SLL）的疗效、影响因素及长期随访结果。

**方法:**

回顾性分析2008年8月至2021年5月期间于江苏省人民医院血液科接受FCR方案一线治疗的68例CLL/SLL患者的疗效、安全性及长期随访结局。

**结果:**

68例患者中女22例、男46例，接受FCR方案治疗时中位年龄为55（47，60）岁。复杂核型（CK）8例（13.1％，8/61），免疫球蛋白重链可变区突变（IGHV-M）20例（32.3％，20/62），del（17p）4例（6.6％，4/61），del（11q）8例（14.8％，8/54）。患者接受FCR方案治疗的中位疗程数为6（4，6）个。患者总反应率为88.2％（60/68），完全缓解率为47.0％（32/68）。中位随访时间为82（59，98）个月，66.2％（45/68）患者出现疾病进展，中位无进展生存（PFS）期为56（21，123）个月，中位总生存期未达到。5年和10年PFS率分别为42.6％（95％ *CI*：31.9％～56.8％）和28.7％（95％ *CI*：19.0％～43.4％）。del（17p）（*HR*＝5.04，95％ *CI*：1.72～14.74，*P*＝0.003）、del（11q）（*HR*＝5.27，95％ *CI*：2.11～13.15，*P*<0.001）、IGHV无突变（*HR*＝4.11，95％ *CI*：1.72～9.79，*P*＝0.001）、CK（*HR*＝3.53，95％ *CI*：1.58～7.85，*P*＝0.002）、β_2_微球蛋白>3.5 mg/L（*HR*＝2.87，95％ *CI*：1.37～6.01，*P*＝0.005）为PFS的不良预后因素，多因素分析显示IGHV无突变为PFS的独立预后因素（*HR*＝8.63，95％ *CI*：1.09～68.40，*P*＝0.042）。16例IGHV-M且无del（17p）及CK的患者中位PFS期达123（58，123）个月，5年PFS率为70.7％（95％ *CI*：49.7％～99.1％），5年后到达PFS平台期，10年内无复发。常见的3～4级不良反应：血液学不良反应（44.1％，30/68），感染（36.7％，25/68），肝功能受损（4.4％，3/68）。25例（56.8％，25/44）患者疾病进展后接受布鲁顿酪氨酸激酶抑制剂单药治疗，中位随访时间为45（26，64）个月，36％（9/25）患者出现疾病进展，中位PFS期为55（27，55）个月。

**结论:**

FCR方案一线治疗可以使IGHV-M且无del（17p）及CK的CLL/SLL患者长期获益。

## Abstract (en)

**Objective:**

To evaluate the efficacy and long-term outcomes of fludarabine, cyclophosphamide, and rituximab（FCR）in treatment-naïve patients with chronic lymphocytic leukemia/small lymphocytic lymphoma（CLL/SLL）.

**Methods:**

Clinical data from 68 CLL/SLL patients treated with FCR at Jiangsu Province Hospital（August 2008–May 2021）were retrospectively analyzed to assess efficacy, safety, and survival outcomes.

**Results:**

Among 68 patients［46 males, 22 females; median age 55（47, 60）years］, 13.1％（8/61）had a complex karyotype, 32.3％（20/62）had immunoglobulin heavy variable region mutated（IGHV-M）type, 6.6％（4/61）had del（17p）, and 14.8％（8/54）had del（11q）. Patients received a median of 6（4, 6）FCR cycles. The overall response rate was 88.2％（60/68）, including 47.0％（32/68）complete remissions. Over a median follow-up of 82（59, 98）months, 66.2％（45/68）experienced disease progression. Median progression-free survival was 56（21, 123）months, while median overall survival was not reached. The 5- and 10-year PFS rates were 42.6％（95％ *CI*: 31.9–56.8％）and 28.7％（95％ *CI*: 19.0–43.4％）, respectively. Poor PFS was associated with del（17p）（*HR*＝5.04, 95％ *CI*: 1.72–14.74, *P*＝0.003）, del（11q）（*HR*＝5.27, 95％ *CI*: 2.11–13.15, *P*<0.001）, IGHV unmutated（IGHV-UM）（*HR*＝4.11, 95％ *CI*: 1.72–9.79, *P*＝0.001）, complex karyotype（CK）（*HR*＝3.53, 95％ *CI*: 1.58–7.85, *P*＝0.002）, β_2_-microglobulin >3.5 mg/L（*HR*＝2.87, 95％ *CI*: 1.37–6.01, *P*＝0.005）. In multivariate analysis, IGHV-UM remained an independent predictor of PFS（*HR*＝8.63, 95％ *CI*: 1.09–68.40, *P*＝0.042）. Sixteen patients with IGHV-M and lacking del（17p）or CK had a median PFS of 123（58,123）months and a 5-year PFS rate of 70.7％（95％ *CI*: 49.7–99.1％）, reaching a plateau after 5 years with no recurrences by 10 years. Common grade 3–4 adverse events included hematologic toxicity（44.1％, 30/68）, infection（36.7％, 25/68）, and liver dysfunction（4.4％, 3/68）. Among 25 patients receiving single-agent BTK inhibitors after FCR progression, median follow-up was 45（26, 64）months; 36％（9/25）experienced disease progression, with a median PFS time of 55（27, 55）months.

**Conclusion:**

First-line FCR provides durable long-term benefits for patients with IGHV-M CLL without del（17p）or CK.

慢性淋巴细胞白血病/小淋巴细胞淋巴瘤（CLL/SLL）是一种共表达CD5和CD23的单克隆成熟小B淋巴细胞增殖性疾病，以单克隆淋巴细胞在外周血、骨髓、淋巴结和脾脏聚集为特征[1]。中位发病年龄为72岁，西方国家的年发病率为4.75/10万，亚洲国家（地区）的年发病率为1.06/10万[2]。以布鲁顿酪氨酸激酶抑制剂（BTKi）与BCL2抑制剂（BCL2i）为代表的小分子靶向治疗改善了CLL的疗效及预后，而E1912、CLL8、CLL10等研究的长期随访数据显示，FCR（氟达拉滨+环磷酰胺+利妥昔单抗）方案一线治疗免疫球蛋白重链可变区突变（IGHV-M）且无TP53基因异常CLL患者，中位随访6～8年，5年无进展生存（PFS）率和总生存（OS）率分别为67％～68％和92％～93％[3]–[5]。本研究对我中心以FCR方案一线治疗的68例CLL/SLL患者进行回顾性分析，探讨该方案在中国患者中的疗效、影响因素及长期随访结果。

## 病例与方法

1. 病例：回顾性分析2008年8月至2021年5月期间于江苏省人民医院血液科就诊的接受FCR方案一线治疗的68例CLL/SLL患者（CLL 53例、SLL 15例）临床资料。纳入的CLL患者初诊时均行外周血或骨髓流式细胞术免疫表型检测，SLL患者初诊时均行淋巴结穿刺或活检病理检测，诊断标准参照国际慢性淋巴细胞白血病工作组2018指南[6]。本研究通过江苏省人民医院伦理委员会审批（批件号：2025-SR-086）。

2. 治疗方案：氟达拉滨25 mg·m^−2^·d^−1^，第1～3天，静脉滴注；环磷酰胺250 mg·m^−2^·d^−1^，第1～3天，静脉滴注；利妥昔单抗375 mg/m^2^，第0天，静脉滴注；28 d为1个疗程。45例患者自第2个疗程起利妥昔单抗改为500 mg/m^2^。患者最多接受6个疗程的FCR方案治疗。

3. 疗效评价：CLL参考《中国慢性淋巴细胞白血病/小淋巴细胞淋巴瘤的诊断与治疗指南（2022年版）》[7]进行疗效评估，SLL患者根据Lugano 2014标准进行疗效评估，包括完全缓解（CR）、部分缓解（PR）、疾病稳定（SD）、疾病进展（PD），总反应率（ORR）为CR率和PR率之和。CLL-IPI评分包含TP53基因突变或缺失、IGHV基因突变状态、β_2_微球蛋白（β_2_-MG）、临床分期及年龄5个指标，根据评分将患者分为低危（0～1分）、中危（2～3分）、高危（4～5分）和极高危（≥6分）。

4. 随访：通过住院病历及门诊病历系统获取患者病历信息，并以电话联系方式进行随访。随访截止时间为2024年11月17日。OS期定义为自诊断之日至患者死亡或随访截止日期的间隔时间。PFS期定义为自患者开始接受治疗之日至任何原因所致复发、死亡或随访截止日期的间隔时间。

5. 统计学处理：统计分析采用R 4.2.2软件进行数据分析。连续性变量以*M*（*Q*_1_，*Q*_3_）表示，分类变量以例（％）表示。连续性变量组间比较采用*t*检验或Mann-Whitney *U*检验。分类变量组间比较采用Fisher精确检验。采用Kaplan-Meier法对生存数据进行估计并绘制生存曲线，利用Log-rank检验对生存数据进行差异分析。中位随访时间采用反式Kaplan-Meier法进行估计。使用Cox回归模型进行PFS的因素分析，将PFS单因素分析中*P*<0.05的相关性因素纳入PFS的多因素分析。*P*<0.05为差异有统计学意义。

## 结果

1. 基线临床特征：如表1所示，68例患者中，女22例（32.4％）、男46例（67.6％），接受FCR方案治疗时的中位年龄为55（47，60）岁。CLL 53例，按照Rai分期，Ⅰ、Ⅱ、Ⅲ和Ⅳ期患者例数分别为13例（19.1％）、18例（26.5％）、5例（7.4％）和17例（25.0％）；按照Binet分期，A、B和C期患者例数分别为4例（5.9％）、30例（44.1％）和19例（27.9％）。SLL 15例，其中Lugano分期ⅢA期、ⅢB期、ⅣA期和ⅣB期分别为2例（2.9％）、1例（1.5％）、6例（8.8％）和6例（8.8％）。携带复杂核型（CK，存在≥3种染色体异常）8例（8/61，13.1％）；IGHV-M 20例（20/62，32.3％），常用的使用片段依次为VH1-69（16.1％，10/62）、VH4-34（14.5％，9/62）和VH3-48（12.9％，8/62）。FISH技术检测del（17p）、del（11q）发生频率分别为6.6％（4/61）、14.8％（8/54）。整合靶向二代测序（NGS）及Sanger测序结果，检测到TP53、NOTCH1、ATM的突变率分别为8.1％（3/37）、5.9％（2/34）和12.5％（3/24）。

**表1:** 68例接受FCR方案一线治疗的CLL/SLL患者临床特征

临床特征	统计值
接受FCR方案治疗时年龄［岁，*M*（*Q*_1_，*Q*_3_）］	55（47，60）
性别［例（％）］	
女	22（32.4）
男	46（67.6）
WBC［×10^9^/L，*M*（*Q*_1_，*Q*_3_）］	21.6（7.6，59.7）
ALC［×10^9^/L，*M*（*Q*_1_，*Q*_3_）］	16.5（4.3，47.7）
HGB［g/L，*M*（*Q*_1_，*Q*_3_）］	128（115，140）
PLT［×10^9^/L，*M*（*Q*_1_，*Q*_3_）］	123（93，171）
LDH［U/L，*M*（*Q*_1_，*Q*_3_）］	208.5（176.0，289.5）
β_2_微球蛋白［mg/L，*M*（*Q*_1_，*Q*_3_）］	3.2（2.3，3.9）
伴B症状［62例，例（％）］	18（29.0）
伴大包块［直径≥5 cm，58例，例（％）］	7（12.1）
伴脾大［60例，例（％）］	30（50.0）
CLL-IPI评分［53例，例（％）］	
1分	16（30.2）
2～3分	18（34.0）
4～5分	14（26.4）
≥6分	5（9.4）
IGHV突变状态［62例，例（％）］	
IGHV-UM	42（67.7）
IGHV-M	20（32.3）
伴CK［61例，例（％）］	8（13.1）
del（17p）［61例，例（％）］	4（6.6）
del（13q）［61例，例（％）］	17（37.8）
del（11q）［54例，例（％）］	8（14.8）
+12［44例，例（％）］	6（13.6）
del（6q23）［34例，例（％）］	4（11.8）
TP53突变［37例，例（％）］	3（8.1）
NOTCH1突变［34例，例（％）］	2（5.9）
SF3B1突变［34例，例（％）］	3（8.8）
ATM突变［24例，例（％）］	3（12.5）
MYD88突变［33例，例（％）］	3（9.1）
EGR2突变［24例，例（％）］	4（16.7）

**注** FCR：氟达拉滨+环磷酰胺+利妥昔单抗；CLL/SLL：慢性淋巴细胞白血病/小淋巴细胞淋巴瘤；ALC：绝对淋巴细胞计数；IGHV：免疫球蛋白重链可变区；IGHV-UM：IGHV未突变；IGHV-M：IGHV突变；CK：复杂核型

2. 治疗反应：68例CLL/SLL患者接受FCR方案治疗的中位疗程数为6（4，6）个，其中接受2、3、4、5和6个疗程治疗的例数分别为3例（4.4％）、6例（8.8％）、15例（22.1％）、4例（5.9％）和40例（58.8％）；95.6％（65/68）患者接受FCR方案治疗≥3个疗程，58.8％（40/68）的患者完成6个疗程。疗程结束时进行疗效评估，ORR为88.2％（60/68）、CR率为47.0％（32/68）、PR率为41.2％（28/68）。3个和6个疗程后可评估患者的CR率分别为24.1％（13/54）和60.0％（24/40）（*P*<0.001），PR率分别为59.3％（32/54）和35.0％（14/40）（*P*＝0.020）。其中，3个疗程后达PR的9例以及达SD的2例患者于6个疗程治疗结束后达到CR。

3. 随访及预后：68例患者中位随访时间为82（59，98）个月，66.2％（45/68）出现疾病进展，其中2例（2.9％）分别于末次治疗后5个月、10个月发生Richter转化［2例均为IGHV无突变（IGHV-UM）、伴有del（17p）］；1例（1.5％）进展时诊断为CLL加速期；4例（5.9％）发生第二肿瘤（乳腺癌、肺癌、食管贲门癌各1例，生发中心来源且非原发CLL同源弥漫大B细胞淋巴瘤1例）；5例（7.4％）死亡，16例失访。中位PFS期为56（21，123）个月，中位OS期未达到。5年和10年的PFS率分别为42.6％（95％ *CI*：31.9％～56.8％）和28.7％（95％ *CI*：19.0％～43.4％），5年和10年的OS率分别为94.7％（95％ *CI*：89.1％～100％）和90.3％（95％ *CI*：82.4％～98.9％）。

对年龄、Rai分期、Binet分期、LDH、β_2_-MG、CK、IGHV突变情况、del（17p）、del（11q）、+12、del（13q）进行PFS预后因素分析，在单因素分析中，del（17p）（*HR*＝5.04，95％ *CI*：1.72～14.74，*P*＝0.003）、del（11q）（*HR*＝5.27，95％ *CI*：2.11～13.15，*P*<0.001）、IGHV-UM（*HR*＝4.11，95％ *CI*：1.72～9.79，*P*＝0.001）、CK（*HR*＝3.53，95％ *CI*：1.58～7.85，*P*＝0.002）、β_2_-MG>3.5 mg/L（*HR*＝2.87，95％ *CI*：1.37～6.01，*P*＝0.005）为PFS的不良预后因素，且IGHV-UM在PFS多因素分析中仍具有预后意义（*HR*＝8.63，95％ *CI*：1.09～68.40，*P*＝0.042）。

基线CLL-IPI评分为低危（1分）、中危（2～3分）、高危（4～5分）及极高危（≥6分）的患者中位PFS期分别为123（58，未达到）、38（23，71）、35（20，58）和15（14，17）个月（*P*<0.001），提示该预后评分系统对该组患者的PFS具有区分和预测价值。进一步对患者IGHV突变状态、del（17p）及CK状态进行PFS预后分析，发现IGHV-M且无del（17p）及CK亚组（16例）能长期获益，中位PFS期为123（58，123）个月，5年PFS率为70.7％（95％*CI*：49.7％～99.1％），5年后到达PFS平台期，10年内无复发；而IGHV-UM合并del（17p）（3例）或合并CK（6例）亚组预后极差［中位PFS期：19.5（17，32）个月和17（14，47）个月］，差异具有统计学意义（*P*<0.001），9例患者随访死亡1例，失访3例。

4. 治疗相关不良反应：治疗期间3～4级血液学不良反应发生率为44.1％（30/68），中性粒细胞减少、血小板减少、贫血分别占23.5％（16/68）、14.7％（10/68）、5.9％（4/68）。非血液学不良反应方面，3级以上感染发生率为36.7％（25/68），其中细菌、真菌、病毒感染分别占26.5％、8.8％、1.5％；3级以上肝功能异常（谷丙转氨酶较接受FCR方案治疗前升高5倍以上）发生率为4.4％（3/68），未发现3～4级的心脏、肾毒性及神经系统异常，未见肿瘤溶解综合征、自身免疫性溶血性贫血、昏迷及瘫痪等并发症。13例（19.1％）患者因4级血液学不良反应而减少1～2个疗程，此外分别有2例和1例患者因4级肺感染和肝功能异常而减少2个疗程，8例患者因治疗期间达CR或PR而个人选择减少1～2个疗程，而另有4例因3个疗程后评估为SD或PD而中断治疗。因4级不良反应减少1～2个疗程化疗的患者和完成6个疗程的患者PFS差异无统计学意义（*P*>0.05）。

5. 接受FCR方案治疗后发生PD后的下一线治疗：45例发生PD的患者中，77.8％（35/45）患者为IGHV-UM型，VH1-69使用片段（25.7％，9/35）最常见；8.9％（4/45）患者携带del（17p）；17.8％（8/45）患者携带CK。44例达到治疗指征而接受下一线治疗，25例选择BTKi单药治疗，10例接受免疫化疗，4例接受BTKi联合BCL2i治疗，3例接受BCL2i单药治疗，1例接受BTKi联合BCL2i联合抗CD20单抗治疗，1例接受BTKi联合化疗。接受BTKi单药治疗的患者中位随访45（26，64）个月，36％（9/25）患者出现疾病进展，中位PFS期为55（27，55）个月。另外，2例Richter转化患者接受R-CHOP（利妥昔单抗联合环磷酰胺+多柔比星+长春新碱+泼尼松）方案治疗，PFS期分别为14和15个月。

## 讨论

国外E1912、CLL10等研究[3],[5]的长线随访数据显示，接受FCR方案一线治疗的IGHV-M且无TP53基因异常CLL患者可长期获益。与FR（氟达拉滨+利妥昔单抗）方案及BR（苯达莫司汀+利妥昔单抗）方案相比[5],[8]，FCR方案有更高的CR率、微小残留病阴性（uMRD）率及PFS率。本研究患者的ORR为88.2％、CR率为47.0％，中位随访6.8年，中位PFS期为56个月，中位OS期未达到，5年PFS率为42.6％，达到与CLL8、E1912临床试验接近的临床疗效[3]–[4]，但本研究纳入患者较少，未来可开展多中心合作扩大样本量调研分析以进一步验证。另外，因检测的例数有限，未能探索本队列中MRD对于PFS的影响。一项来自德国CLL研究小组的关于接受免疫化疗患者的研究显示[9]，外周血MRD阳性和骨髓MRD阳性都与较差的PFS独立相关，提示在完成免疫化疗后，可以利用uMRD评估PFS预后[10]。本研究包含Richter转化在内的第二肿瘤发生率为10.3％，与CLL8临床研究中13.1％的发生率接近[4]，提示FCR方案在治疗初治患者中显示出良好的疗效。但FCR方案有较强的血液学毒性等不良反应，导致治疗的耐受性降低，CLL8临床研究中FCR组3～4级血液学不良反应的发生率为56％；3～4级感染发生率为25％[11]。本队列治疗期间3～4级血液学不良反应发生率为44.1％，3级以上感染发生率为36.7％。在对不良反应管理措施方面，本队列13例患者因4级血液学不良反应而减少1～2个疗程，此外分别有2例和1例患者因4级肺感染和肝功能异常而减少2个疗程，经预后因素分析，减少1～2个疗程化疗的患者未缩短PFS期，完成6个疗程化疗的患者可获得较高的CR率。本队列中对于其他出现3级以上血液学不良反应及感染的患者，在患者耐受情况下继续予FCR方案治疗，并采取对症治疗处理，包括输血成分或短/长效重组人粒细胞刺激因子注射液、积极抗感染等。所有不良反应经对症处理后均恢复。

预后方面，CLL8临床研究结果显示，IGHV-UM、del（17p）与较短的PFS期相关，del（17p）亚组中位PFS期为11.2个月，IGHV-UM亚组中位PFS期为42.8个月[4]。另一项接受一线FCR方案治疗的CLL患者的回顾性研究显示，CK与较短的PFS期相关（21个月）[12]。本队列结果显示del（17p）、IGHV-UM、CK为PFS的不良预后因素，且IGHV-UM为PFS的独立预后因素。此外，CLL8临床研究还显示了TP53、NOTCH1、SF3B1基因突变与较短的PFS期相关，其5年PFS率分别为15.3％、26.7％和31.1％[8]。因本队列中检测患者有限，突变阳性患者例数少，未能针对TP53、NOTCH1基因突变等对于PFS的影响进行差异性分析。CLL-IPI评分系统是免疫化疗时代针对CLL的预后评分系统，可预测免疫化疗的PFS和OS结果[13]–[14]，本队列验证了CLL-IPI风险分组对FCR方案一线治疗患者的PFS的区分能力。值得注意的是，经长线随访，IGHV-M且无del（17p）及CK的患者能长期获益。既往临床研究也报道了年轻（<65岁或70岁）、IGHV-M、不伴del（17p）的CLL患者一线应用FCR方案可获得较好的生存，然而FCR方案无法改变IGHV-UM的预后不良结局[15]。进入新药时代后，BTKi可以克服IGHV-UM的不良预后影响[16]。E1912临床研究中，IR（伊布替尼+利妥昔单抗）亚组在PFS和OS上均优于FCR亚组，尤其对于IGHV-UM的CLL患者（5年PFS率：75％对33％）[3]。一项iFCG（伊布替尼+氟达拉滨+环磷酰胺+奥妥珠单抗）方案治疗IGHV-M且无del（17p）及CK的初治CLL临床研究中[17]，患者经3个周期iFCG方案治疗后，骨髓uMRD率（CLL细胞占白细胞的比例低于1/10 000）可达到98％，6年PFS率为92％。cwCLL-001临床研究结果也提示oFCG（奥布替尼+氟达拉滨+环磷酰胺+奥妥珠单抗）方案治疗具有不良预后特征的初治CLL患者可达到快速、深度的分子缓解[18]。综上，与小分子靶向药物BTKi的联合治疗可以达到快速和深度的分子缓解、缩短疗程以及降低第二肿瘤发生率。对年轻的IGHV-M且无del（17p）及CK的初治CLL患者，在经济效益上，仍可考虑选择FCR方案或者在MRD指导下选择BTKi联合FCR/G方案的有限疗程。

在接受FCR治疗后复发/进展患者中，55.6％（25/45）患者在下一线接受单药BTKi治疗，中位PFS期为55个月。在PCYC-1102临床试验中，伊布替尼治疗复发/难治患者［34％携带del（17p），37％患者携带CK，78％为IGHV-UM］也能获得52个月的中位PFS期、34％的7年PFS率，较免疫化疗效果有明显提高[19]–[20]。对于FCR方案治疗后PD患者，可推荐各类小分子靶向药物作为下一线的治疗选择。

综上，本中心使用FCR方案治疗的患者队列达到与CLL8临床研究中FCR亚组接近的疗效[4]，且本队列显示FCR方案一线治疗可以使IGHV-M且无del（17p）及CK的患者长期获益。在新药时代，FCR方案可作为年轻、体能状态良好、无TP53基因异常、无CK且IGHV-M的CLL/SLL患者固定有限周期的治疗方案，并可尝试在此基础上与新药联合的有限疗程治疗。

## References

[1] Rai KR, Jain P (2016). Chronic lymphocytic leukemia (CLL)-Then and now[J]. Am J Hematol.

[2] Bassig BA, Au WY, Mang O (2016). Subtype-specific incidence rates of lymphoid malignancies in Hong Kong compared to the United States, 2001-2010[J]. Cancer Epidemiol.

[3] Shanafelt TD, Wang XV, Hanson CA (2022). Long-term outcomes for ibrutinib-rituximab and chemoimmunotherapy in CLL: updated results of the E1912 trial[J]. Blood.

[4] Kutsch N, Giza A, Robrecht S (2024). Fludarabine, cyclophosphamide, and rituximab as first-line treatment in patients with chronic lymphocytic leukemia: a long-term analysis of the German CLL Study Group (GCLLSG) registry[J]. Eur J Haematol.

[5] Kutsch N, Bahlo J, Robrecht S (2020). Long term follow-up data and health-related quality of life in frontline therapy of fit patients treated with FCR versus BR (CLL10 trial of the GCLLSG)[J]. Hemasphere.

[6] Hallek M, Cheson BD, Catovsky D (2018). iwCLL guidelines for diagnosis, indications for treatment, response assessment, and supportive management of CLL[J]. Blood.

[7] 中国抗癌协会血液肿瘤专业委员会, 中华医学会血液学分会, 中国慢性淋巴细胞白血病工作组 (2022). 中国慢性淋巴细胞白血病/小淋巴细胞淋巴瘤的诊断与治疗指南(2022年版)[J]. 中华血液学杂志.

[8] Fischer K, Bahlo J, Fink AM (2016). Long-term remissions after FCR chemoimmunotherapy in previously untreated patients with CLL: updated results of the CLL8 trial[J]. Blood.

[9] Kovacs G, Robrecht S, Fink AM (2016). Minimal residual disease assessment improves prediction of outcome in patients with chronic lymphocytic leukemia (CLL) who achieve partial response: comprehensive analysis of two phase III studies of the German CLL Study Group[J]. J Clin Oncol.

[10] 黄 燕姗, 邱 录贵, 易 树华 (2023). 微小残留病监测在B细胞淋巴瘤中的研究进展[J]. 白血病·淋巴瘤.

[11] Hallek M, Fischer K, Fingerle-Rowson G (2010). Addition of rituximab to fludarabine and cyclophosphamide in patients with chronic lymphocytic leukaemia: a randomised, open-label, phase 3 trial[J]. Lancet.

[12] Cavallari M, Cavazzini F, Bardi A (2018). Biological significance and prognostic/predictive impact of complex karyotype in chronic lymphocytic leukemia[J]. Oncotarget.

[13] (2016). An international prognostic index for patients with chronic lymphocytic leukaemia (CLL-IPI): a meta-analysis of individual patient data[J]. Lancet Oncol.

[14] 朱 华渊, 王 莉, 乔 佳 (2018). CLL-IPI评分系统在中国慢性淋巴细胞白血病患者中的预后评估价值[J]. 中华血液学杂志.

[15] Ahn IE, Tian X, Albitar M (2018). Validation of clinical prognostic models and integration of genetic biomarkers of drug resistance in CLL patients treated with ibrutinib[J]. Blood.

[16] Burger JA, Barr PM, Robak T (2020). Long-term efficacy and safety of first-line ibrutinib treatment for patients with CLL/SLL: 5 years of follow-up from the phase 3 RESONATE-2 study[J]. Leukemia.

[17] Jain N, Thompson P, Jain A (2023). Ibrutinib, fludarabine, cyclophosphamide and obinutuzumab (iFCG) for firstline treatment of patients with CLL with mutated IGHV and without del(17p)/TP53 mutation: six-year follow-up analyses[J]. Blood.

[18] Zhu H, Miao Y, Qin S (2024). Orelabrutinib, fludarabine, cyclophosphamide, and obinutuzumab (OFCG) for first-line treatment of chronic lymphocytic leukemia: a multicenter, investigator-initiated study (cwCLL-001 study)[J]. Blood.

[19] Ahn IE, Tian X, Wiestner A (2020). Ibrutinib for chronic lymphocytic leukemia with TP53 alterations[J]. N Engl J Med.

[20] Byrd JC, Furman RR, Coutre SE (2020). Ibrutinib treatment for first-line and relapsed/refractory chronic lymphocytic leukemia: final analysis of the pivotal phase Ib/II PCYC-1102 study[J]. Clin Cancer Res.

